# The spatial and temporal effect of electrochromic windows on indoor and human microbiome in an inpatient hospital

**DOI:** 10.1017/ash.2024.344

**Published:** 2024-10-25

**Authors:** Man In Lam, Kelsey Gleason, Allen B. Repp, Sam Yeo, Kinga Vojnits, Piers MacNaughton, Sepideh Pakpour

**Affiliations:** 1 Faculty of Applied Science, School of Engineering, University of British Columbia, Kelowna, BC, Canada; 2 Department of Biomedical and Health Sciences, University of Vermont, Burlington, VT, USA; 3 Department of Medicine, The Larner College of Medicine at the University of Vermont, Burlington, VT, USA; 4 Department of Environmental Health, Harvard T.H. Chan School of Public Health, Boston, MA, USA

## Abstract

**Objective:**

Improving the hospital environment and developing novel disinfection strategies are critical for infection control in healthcare settings. In this study, we explored the effects of electrochromic (EC) windows on indoor and patient microbiome in an inpatient hospital.

**Patient and setting:**

Hematology-Oncology patients at the University of Vermont Medical Center

**Methods:**

We conducted a prospective study in ten occupied patient rooms. Five of the patient rooms had active EC windows that tint dynamically to control for heat and glare, and the other five rooms had deactivated EC windows that simulated traditional windows and blinds. Samples were collected one day before patient admission as baseline and on the 1st, 3rd, and 5th day of the patient stay. Total bacterial abundance and bacterial community structure were determined through quantitative PCR and 16s rRNA Illumina MiSeq sequencing, respectively.

**Results:**

Patient rooms with active EC windows had significantly lower light intensity and temperature than traditional patient rooms with blinds. The absolute bacterial abundance and diversities on windows were significantly lower in rooms with EC windows and the bacterial composition changed after one day EC window activation. Compared to baseline, relative abundance of the *Staphylococcus* genus was significantly lower on EC window surface during the five-day experiment. In contrast, the air microbiome was more diverse in rooms with EC windows.

**Conclusion:**

Active electrochromic (EC) windows in patient rooms result in lower light intensity and temperature, reduced bacterial abundance and diversities on window surfaces, and a more diverse air microbiome, informing future healthcare design.

## Introduction

Reducing healthcare-associated infections (HAIs) is a major focus for hospitals. One in ten patients acquire a HAI during their hospital stay, leading to over 99,000 premature deaths in the United States (US) each year.^1^ Hospitalized patients are more likely to carry pathogenic microbes and are more susceptible to infections than the public.^2,3^ Nosocomial pathogens can also survive months on dry surfaces such as medical devices, venetian blinds, and personal tablets,^4^ thus contact with these contaminated surfaces may pose a threat to patients. Hospital building design features may alter the environmental microbiome and may offer a novel approach to addressing HAIs.

Many disinfection strategies have been developed to reduce the likelihood of pathogens in hospitals.^2,5^ Recently, growing interests have been given to the photoinactivation properties of high-energy violet-blue light,^6,7^ as light at 400–420nm does not appear to cause severe damage to mammalian cells and can be used without disrupting clinical flow.^8,9^ Previously in a laboratory-scale study, we compared how sunlight passing through two different windows—traditional glass windows covered with blinds and electrochromic (EC) glass windows that adjust their tint in response to the sun—affected the growth of harmful bacteria responsible for most HAIs, including *methicillin-resistant Staphylococcus aureus* (*MRSA*), *Pseudomonas aeruginosa, Klebsiell*a *pneumoniae*, and *Escherichia coli*.^10^ Results showed that EC window glass, which transmitted 10 times more short-wavelength, high-energy daylight (400–420nm) compared to regular window glass, reduced surface bacteria in both its clear and tinted states relative to the blinds condition.^10^ It has also been shown that application of such windows can significantly improve occupants’ comfort and productivity by auto-adjusting the darkness of windows tints, reducing unwanted thermal transfer, and creating a smart dynamic indoor environment.^11–13^ However, can we harness these benefits in a real building? In this prospective, randomized study, we sought to understand the impact of EC windows on environmental conditions and the surface and air microbiome in patient-occupied rooms in a hospital building.

## Methods

### Setting

Biological and environmental data collection was conducted in one inpatient building (the Miller Building) at the University of Vermont Medical Center (Burlington, VT, USA) in April and May 2021. Ten west-facing patient rooms on the 5^th^ floor of the Miller Building were included in this study. Sample collection started one day before patient admission (T0: baseline), the day of patient admission to the hospital (T1: admission), three days after patient admission (T2) and five days after admission (T3). At T0, all rooms had deactivated electrochromic (EC) windows. From T1 to T3, windows in five of the patient rooms remained deactivated with traditional blinds (sheer fabric roller shade), simulating traditional windows and blinds (Regular Room). The other five rooms had active EC windows (View Inc, USA) without blinds (EC Window Room). The EC windows used in this study have been described previously by Lam et al.^10^ and can dynamically control indoor daylight levels based on current outdoor solar radiation without the need of conventional roller blinds. The main difference in the indoor light spectrum between Regular Room and EC Window Room is in the violet-blue light spectrum (400-420nm), as EC windows transmit 10 times more blue light into buildings compared to traditional windows with blinds.^10^

### Environmental data collection

To assess the indoor environment quality, an environmental sensor (Awair Omni, California) was installed in each patient room to record the light intensity, temperature, relative humidity (RH), carbon dioxide (CO_2_) and particulate matter 2.5 (PM2.5) level throughout all experiment days (T0 through T3, continuously). All conditions were logged at 15-minute intervals. No chemical or other cleaning products were used during the sample collection period for each room, although basic cleaning procedures such as floor sweeping, and trash removal were conducted.

### Environmental microbiologic sample collection

Surface samples were collected from windows, blinds, and air ducts using sterile BD BBL^TM^ CultureSwab (Fisher Scientific, Canada). Details of surface sample collection can be found in the Supplemental Material. Air samples were collected onto a clean polytetrafluoroethylene (PTFE) (0.3µm, 37mm) filter using SKC AirChek Touch Pump (SKC Inc, USA) at a flow rate of 3.5L/min for 180 minutes. All samples were stored at –80°C freezer immediately upon collection for further analysis.

### Patient microbiologic sample collection

All participants provided informed consent at the time of recruitment and the study protocol was approved by the institutional review board at the University of Vermont Medical Center (study ID: IRB00001176). Patients ≥18 years old who were admitted to the Hematology-Oncology service with an anticipated inpatient length of stay greater than three days were recruited for study participation. Exclusion criteria included individuals on contact isolation or airborne isolation precautions and those who had cognitive impairment or dementia. As part of the consent process, participants assigned to Regular Rooms agreed to leave the blinds in the assigned position.

Biological samples were collected from each patient’s palm and saliva. Palm samples were collected by swabbing the patient’s palm horizontally, vertically in a zigzag pattern and in between fingers. For saliva samples, patients rinsed their mouth with water prior to sample collection, and approximately 2mL of the saliva was collected into a sterile 15mL falcon tube. All samples were stored at –80°C freezer immediately upon collection for further analysis.

### DNA extraction & 16s rRNA Illumina MiSeq sequencing

Total DNA from swabs and air filters was extracted using the DNeasy PowerSoil Kit (Qiagen, USA). Saliva samples were extracted using QIAamp DNA Microbiome Kit (Qiagen, USA). After sample preparation, the extracted DNA was sent for Illumina MiSeq sequencing and the V3V4 regions of 16s rRNA were amplified (341F-806R) to determine the bacterial community structures. The details of DNA extraction, sample sequencing preparation and data processing can be found in the Supplemental Material.

### Real-time quantitative polymerase chain reaction (qPCR)

To quantify the absolute bacterial abundance, qPCR was performed on CFX Opus 96 Real-Time PCR System (Bio-Rad, Canada) based on the protocol by Shrestha et al. targeting the V4 region of 16s rRNA (515F-806R).^14^ Details of qPCR conditions can be found in the Supplemental Material. Limit of detection (LOD) of qPCR machine was predetermined to be 35 copies per μL (Figure S1). Results below this threshold were reported as below LOD.

### Statistical analysis and bioinformatics

Python version 3.8.13 was used for statistical analysis of environmental data and qPCR results. Results were compared between the Regular Room and EC Window Room groups using either a t-test or Mann-Whitney test. Details can be found in Supplemental Information.

R version 4.2.0 was used for microbial community analysis. Principal coordinate analysis (PCoA) and permutational multivariate analysis of variance (PERMANOVA) were used to assess the similarity and dissimilarity of bacterial community structures. Figures were generated using GraphPad Prism version 9.1.1 (Dotmatics, MA, USA); numerical data were presented as mean ± standard error of the mean (SEM).

## Results

### Environmental conditions

**Figure 1.**
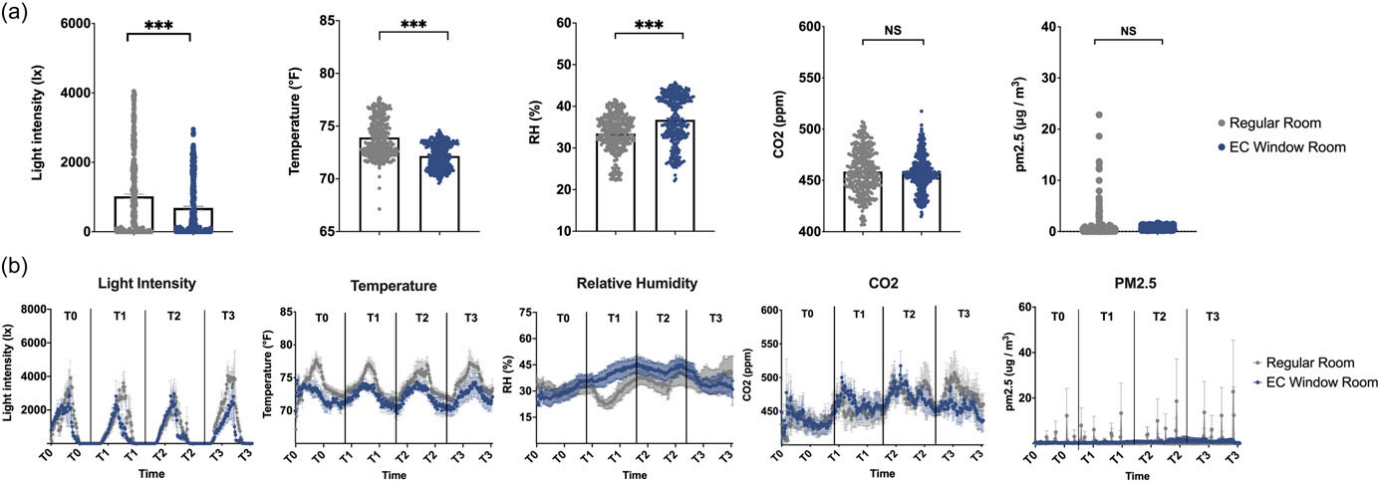
Environmental conditions in Regular Rooms (gray) and EC Window Rooms (blue). (a) Box plots of light intensity, temperature, relative humidity (RH), CO_2_, and PM2.5 concentration. Data show the mean ± SEM. Statistical analysis compared results between Regular Rooms and EC Window Rooms (*, *P <* 0.05; **, *P* < 0.01; ***, *P* < 0.001). (b) Line graph of environmental conditions from T0 to T3. Data show mean ± SEM.

Patient rooms with EC windows had significantly lower light intensity and temperature over the five-day experiment (Mann-Whitney test: light intensity *P* < 0.001, temperature *P* < 0.001, Figure 1). The mean light intensities were 977lx and 683lx; mean temperatures were 73.8°F and 71.9°F (Regular Rooms and EC Window Rooms, respectively for both). Significantly higher humidity was detected in the EC Window Rooms (mean RH: 35.1%) compared to the Regular Rooms (mean RH: 33.2%) (Mann-Whitney test: *P* < 0.001, Figure 1). No significant difference was found in the CO_2_ and PM2.5 concentrations overall between the Regular Room and EC Window Room groups (Figure 1).

### Patient microbiome and indoor environment microbiome: bacteria source tracking

**Figure 2.**
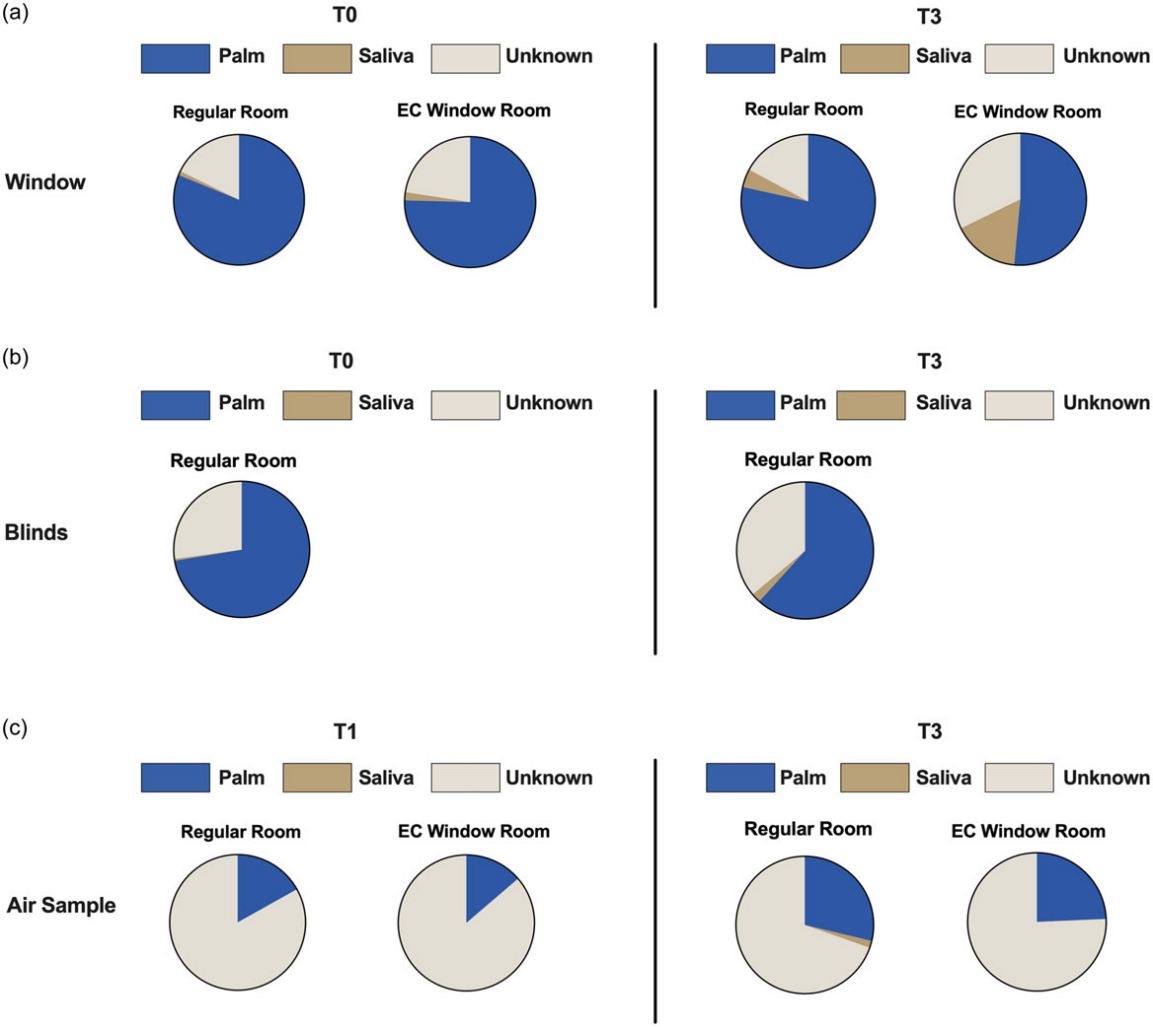
Bacteria source tracking analysis estimated the relative contribution of patient microbiome as sources for indoor environment microbiome. (a) Relative contribution of patient’s microbiome as sources for window sample’s microbiome at T0 on the left and at T3 on the right. Pie chart shows the proportion of patient’s palm microbiome in blue, saliva microbiome in brown, and unknown microbiome in white. (b) Relative contribution of patient’s microbiome as sources for blind microbiome at T0 (left) and at T3 (right). (c) Relative contribution of patients’ microbiome as sources for air sample’s microbiome at T0 (left) and at T3 (right).

We performed a Bayesian-based source tracking analysis using the SourceTracker 2 classifier^15^ to determine the contribution of the patient microbiome as a source of indoor microbiome communities, as occupants are known to impact the built environment microbiome.^16,17^ Results showed that majority of the microbiota found on windows and blinds originated from patients (Figure 2a,b). At T0, 75.4% & 81.2% of the window microbiome originated from patient’s palm, and 1% & 2% were from saliva (EC Window Rooms and Regular Rooms, respectively for both) (Figure 2a). After five days of patient stay (T3), the average contribution of saliva microbiome on EC window surface increased to 16.2% and palm microbiome decreased to 51.5% (Figure 2a). However, this change was not seen in Regular Rooms, most likely due to the use of blinds. Similar results were found on blind surfaces (Regular Rooms), with an average of 72.4% relative contribution from patients’ palm microbiome at T0 and 61.7% at T3 (Figure 2b). Taxa boxplots also showed that skin-associated bacteria were abundantly found on surfaces such as windows, blinds, and air ducts (Figure S2).

Interestingly, microbiota found in air samples mainly originated from unknown sources (Figure 2c). The average relative contribution of unknown microbiome was 83.1% & 86.3% at T0, and 69.6% & 75.7% at T3 (Regular Rooms and EC Window Rooms, respectively). Patient microbiome had minimal contribution to air microbiome (Figure 2c), with nearly 0% originating from saliva at both T1 and T3 in both types of patient rooms.

### Association between window types and the bacterial community structures of environmental samples

#### Absolute bacterial abundance

**Figure 3.**
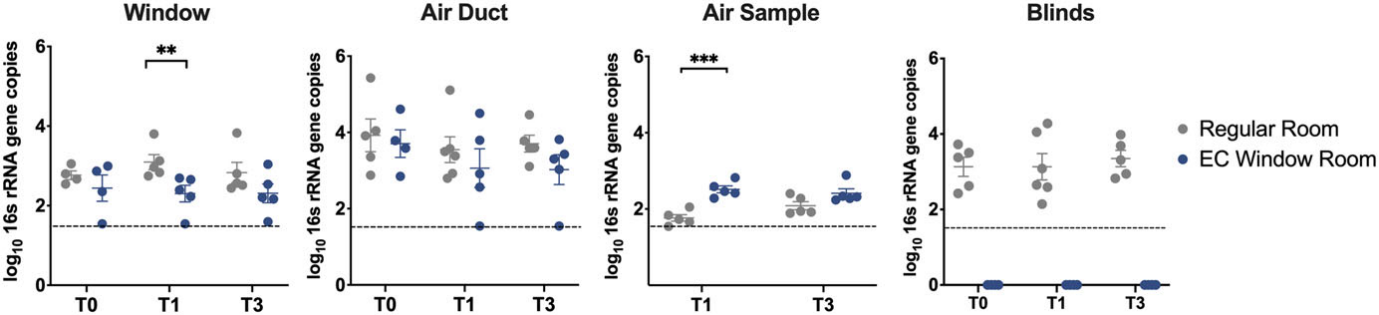
Association between window types and the absolute bacterial abundance of indoor environment samples. Box plots show the copies of 16s rRNA genes found in window, air duct, air sample and blinds. Data show the mean ± SEM. The Regular Room group is shown in gray, and the EC Window Room group is shown in blue. Dashed line shows the LOD of qPCR (35 copies of genes). Statistical analysis compared results between Regular Rooms and EC Window Rooms (*, *P* < 0.05; **, *P* < 0.01; ***, *P* < 0.001).

The total bacterial abundance was quantified using 16S rRNA qPCR. Very low bacterial abundance was detected on window surfaces and in the air. Four window samples (n = 38) and one air sample (n = 19) had lower number of gene copies than the qPCR LOD (35 copies of gene) (Figure 3). In contrast, high bacterial abundance was detected on blinds (only in use in Regular Room group), with all 19 samples above the LOD of qPCR (Figure 3). For EC Window Rooms, a significantly lower absolute bacterial biomass was observed on window surfaces compared to Regular Rooms after one day EC window activation (Mann-Whitney test at T1: *P =* 0.008, Figure 3). In contrast, air samples in EC Window Rooms had significantly higher bacterial abundance compared to Regular Rooms at T1 (two sample t-test: *P <* 0.001, Figure 3). The total bacterial abundance at T3, however, was the same between EC Window Rooms and Regular Rooms on the window surface and in the air.

#### Microbial diversity and community composition

**Figure 4.**
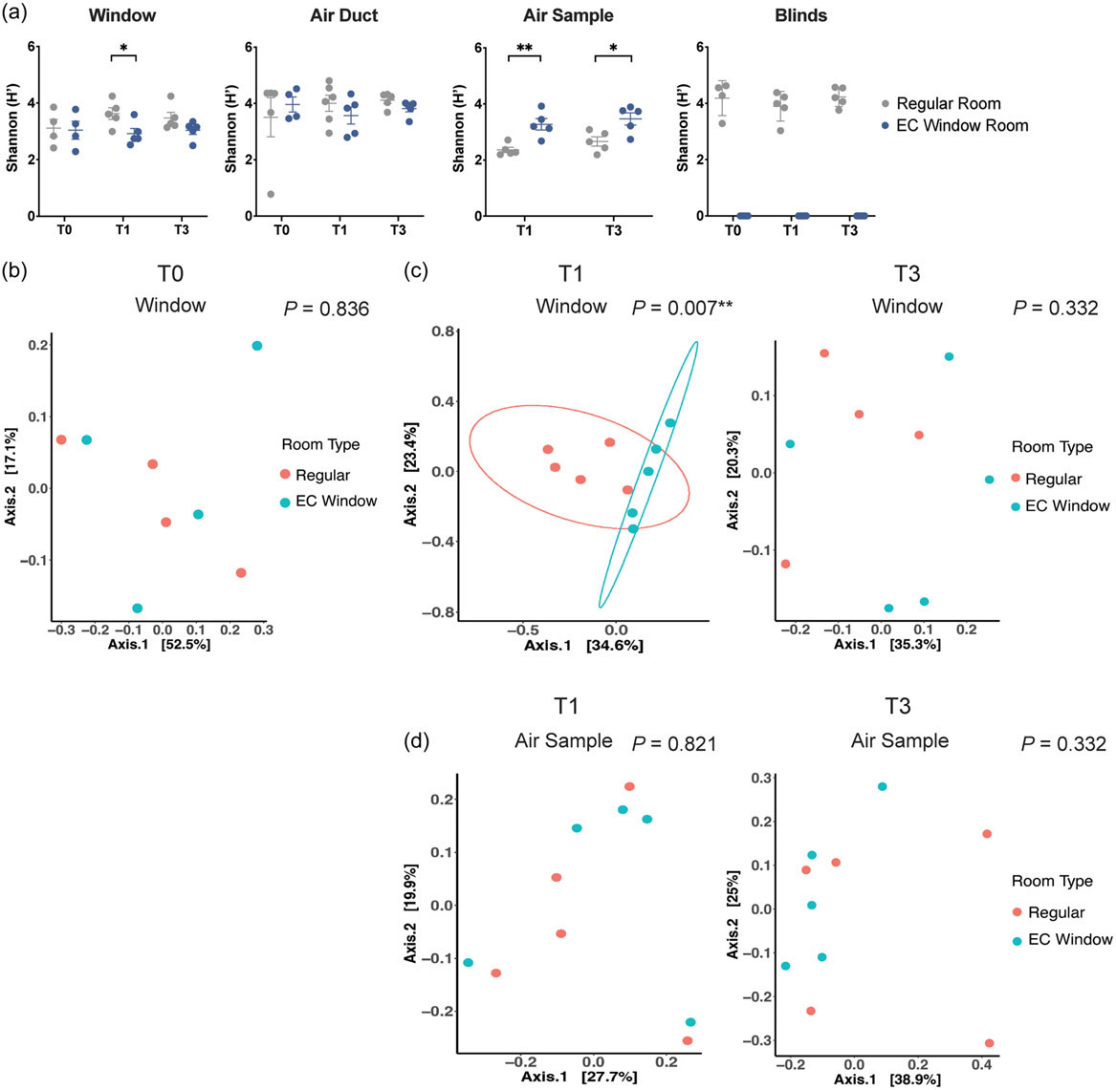
Association between window types and the microbial diversity and community composition on indoor environmental samples. (a) Alpha diversity of environmental samples. Boxplots show the Shannon indices of window, air duct, air, and blinds samples. Data show the mean ± SEM. The Regular Room group is shown in gray, and the EC Window Room group is shown in blue. Statistical analysis compared results between Regular Room and EC Window Room (*, *P* < 0.05; **, *P* < 0.01; ***, *P* < 0.001). (b) Principal coordinates analysis (PCoA) of beta diversity of window samples at T0 based on weighted UniFrac dissimilarities. Regular Room is colored in red, and EC Window Room is colored in green. Significance is determined by permutational multivariate analysis of variance (PERMANOVA) with 999 permutations for room type and denoted at the corner of each PCoA (*, *P* < 0.05; **, *P* < 0.01; ***, *P* < 0.001). (c) PCoA of beta diversity of window samples at T1 and T3 based on weighted UniFrac dissimilarities. Ellipses are drawn at 95% confidence intervals for each room type. (d) PCoA of beta diversity of air samples at T1 and T3 based on weighted UniFrac dissimilarities.

The association between window types and microbial community (alpha- and beta-diversities) was analyzed with the 16s rRNA sequencing results. For alpha diversity, the Shannon index was calculated to account for both the richness and evenness of observed taxa.^18^ Results showed that Shannon diversity on window surfaces became significantly lower in EC Window Rooms compared to Regular Rooms after one day of EC window activation (T1) (Two Sample *t*-test: *P =* 0.033, Figure 4a). In contrast, the Shannon diversity in air samples was significantly higher in the EC Window Rooms at both T1 and T3 (Two Sample t-test; *P =* 0.003, *P* =0.02, respectively; Figure 4a).

For beta diversity, among the three tested distance matrices (Figure S3), weighted UniFrac distance was chosen to assess the bacterial community structures because it considers broad-scale phylogenetic differences between microbial communities and has less bias toward shared distribution of most abundant taxa.^19^ Bacterial compositions on window surfaces were significantly different between Regular Rooms and EC Window Rooms after one day of EC window activation (PERMANOVA between room types at T1: *P* = 0.007, F = 2.736, *R* ^2^= 0.255; Table S1, Figure 4c). However, the difference was not observed at T3 after five days EC Window activation. In contrast, the bacterial compositions of air duct and air filter samples were similar in the Regular Rooms and EC Window Rooms at T1 or T3 (Figure S3, Figure 4d).

### Association between window types and the relative abundance of the ESKAPE pathogens genus

**Figure 5.**
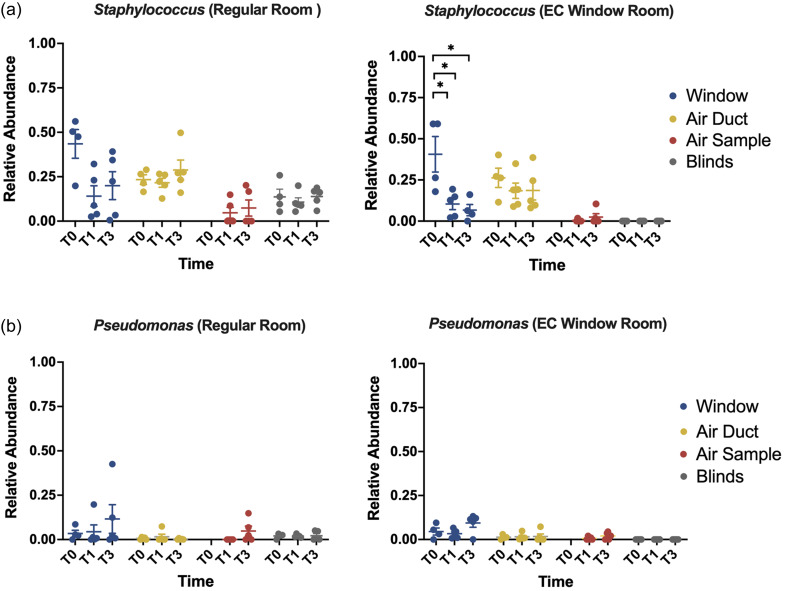
(a) Relative abundance of *Staphylococcus* in indoor environment samples at T0, T1 and T3. The Regular Room group is shown on the left and the EC Window Room group is shown on the right. Window is shown in blue; air duct is shown in yellow; air sample is shown in red, and blind is shown in gray. Boxplots show the mean ± SEM. Statistical analysis compared results between T0 to T1 and to T3 separately (*, *P <* 0.05; **, *P* < 0.01; ***, P < 0.001). (b) Relative abundance of *Pseudomonas* in indoor environment samples. Boxplots show the mean ± SEM.

*Enterococcus faecium, S. aureus, K. pneumoniae, Acinetobacter baumannii, P. aeruginosa,* and *Enterobacter spp*. (ESKAPE) are six multi-drug resistant pathogens that can cause severe HAIs.^20^ Majority of the bacteria in genera ESKAPE were detected in very low abundance (Figure S4), except for *Staphylococcus* and *Pseudomonas* (Figure 5). A significantly lower abundance of *Staphylococcus* genus was observed on windows in EC Window Rooms from baseline (T0) to T1 and to T3 (Pairwise Tukey HSD test: T0 vs T1: *P* = 0.001, T0 vs T3: *P* = 0.007; Figure 5a). The same effects however were not observed in Regular Rooms.

### Association between window types and patient microbiome

**Figure 6.**
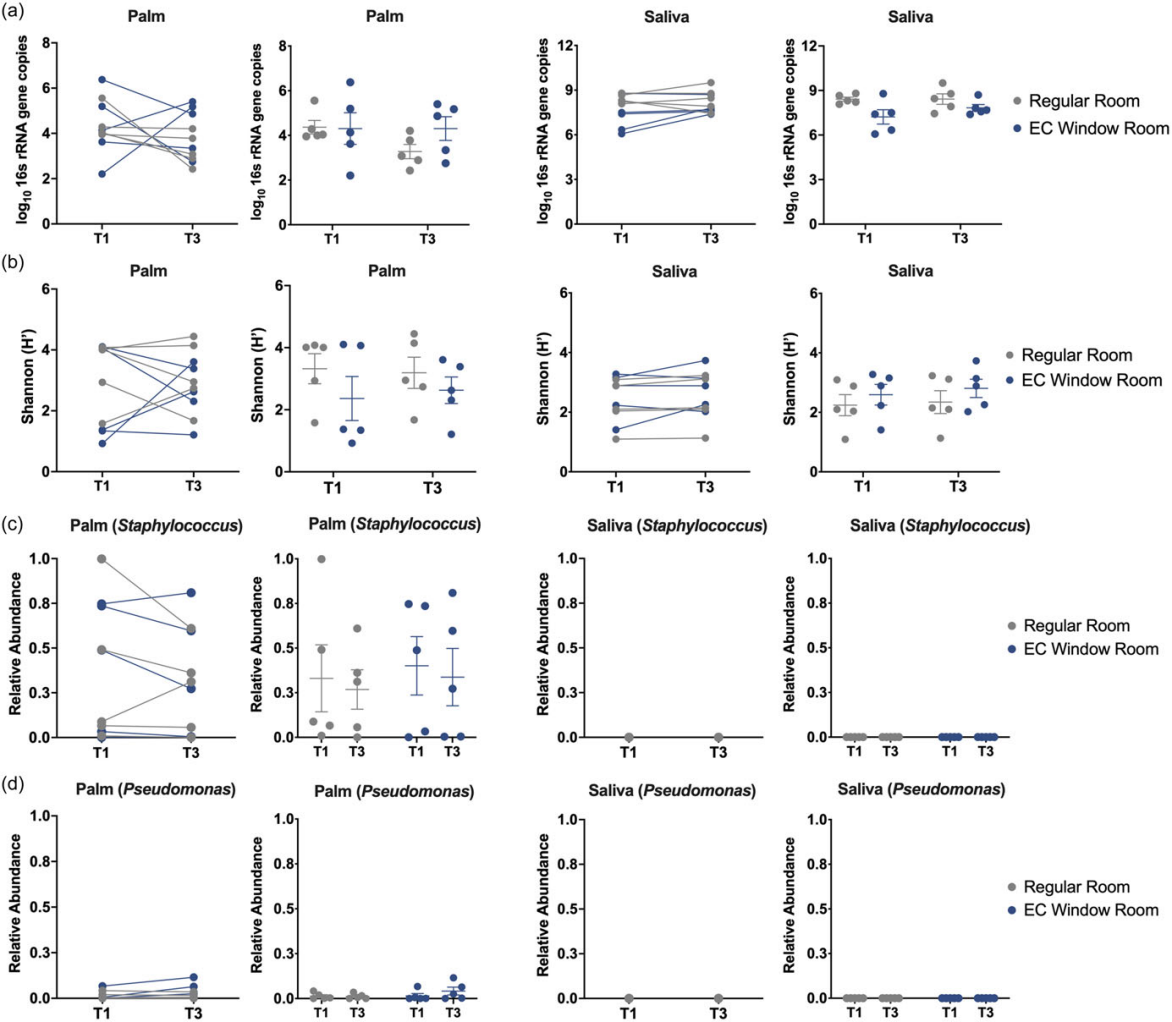
Association between window types and patient microbiome. (a) Box plots show the absolute bacterial abundance of patient’s palm (left) and saliva (right) samples at T1 and T3. The Regular Room group is shown in gray, and the EC Window Room group is shown in blue. Data show the mean ± SEM. Line graph shows the changes of absolute bacterial abundance of individual patient samples from T1 to T3. (b) Shannon diversity of palm (left) and saliva (right) samples at T1 and T3. (c) Relative abundance of *Staphylococcus* in palm (left) and saliva (right) samples at T1 and T3. (d) Relative abundance of *Pseudomonas in* palm (left) and saliva (right) samples at T1 and T3.

The absolute bacterial abundance and Shannon index of patient palm and saliva samples were not significantly different between EC Window Rooms and Regular Rooms at T1 nor T3 (Figure 6a,b). Line graphs showed the total bacterial abundance and microbial diversities of palm and saliva microbiome did not increase significantly from T1 to T3 (Figure 6a,b), suggesting their microbiome remained relatively stable over the first five days of patient stay. The relative abundance of *Staphylococcus* and *Pseudomonas* in palm and saliva samples were also not significantly different between the room types at any time (Figure 6c,d), suggesting EC windows did not have a profound effect on them over the five-day study period.

## Discussion

This study found that window types were associated with environmental microbiome patterns on window surfaces and in the air of patient-occupied hospital rooms. Specifically, we found that rooms with active EC windows had significantly lower total bacterial abundance on the window surface compared to those with traditional windows and blinds and the bacterial community structures became significantly different after one day of EC window activation. Our previous laboratory study showed that EC window allows greater transmission of short-wavelength, high-energy daylight (400–420nm) and can significantly reduce the growth of surface-borne bacteria pathogens.^10^ We therefore hypothesize that light transmission was the primary factor mediating the observed differences, although other environmental factors may have contributed to the observed changes, as environmental data showed that temperature and RH were also significantly different between the two types of patient rooms.

It is noteworthy that the association of EC windows and window surface microbiome, however, was not observed after five-day EC window activation (T3). This might be explained by the role of occupants. Bacteria source tracking results revealed that the patient’s palm was the main contributor to the microbiome found on the window surfaces. The colonization of patient’s microbiome on indoor surfaces has been addressed by many studies;^16–18,21^ Lax et al. (2017) showed that the environmental surfaces of a newly opened hospital were entirely occupied by the microbiota of patients within 24 hours of admission.^18^ Skin-associated microbiome can accumulate on indoor surfaces over five days (Figure S2), diminishing the effects of EC windows, which was highly likely considering no cleaning activities were conducted during the study period.

Air samples in the EC Window Rooms showed higher absolute bacterial abundance and microbial diversities. We speculate that the higher RH in EC Window Rooms could account for the observed differences: Kokubo et al. found that the DNA concentrations of indoor air of a traditional Japanese household were positively correlated with the humidity levels.^22^ Other environmental factors such as temperature, CO_2_ concentration, and PM2.5 can also affect airborne bacterial communities which are potentially confounding factors that cannot be controlled in this hospital.^19,21–23^ Additionally, the air microbiome was mainly from unknown sources, an indication that the outdoor air microbiome dominates the indoor air in well-ventilated spaces.^16,24^

One of the main advantages of using the EC windows was the removal of shades, as blinds harbored significant number of bacteria with all 19 samples detected by qPCR. The porous material can entrap microorganisms and serve as a reservoir for pathogens. Studies have shown that 92% of the curtains in hospitals are contaminated with *MRSA* within one week of being cleaned.^25,26^ Pathogens retained on the blinds can be re-suspended into the environment when patients lower, raise, or interact with the blinds, potentially facilitating disease transmission.^26^ In comparison, window surfaces harbored significantly lower absolute bacterial abundance (4 samples below qPCR LOD), which can be accounted for by the low nutrient density and smooth surface of glass which does not support bacteria attachment.^27^ Window glass is also easily accessible for frequent cleaning and disinfection.^2^

**Figure 7.**
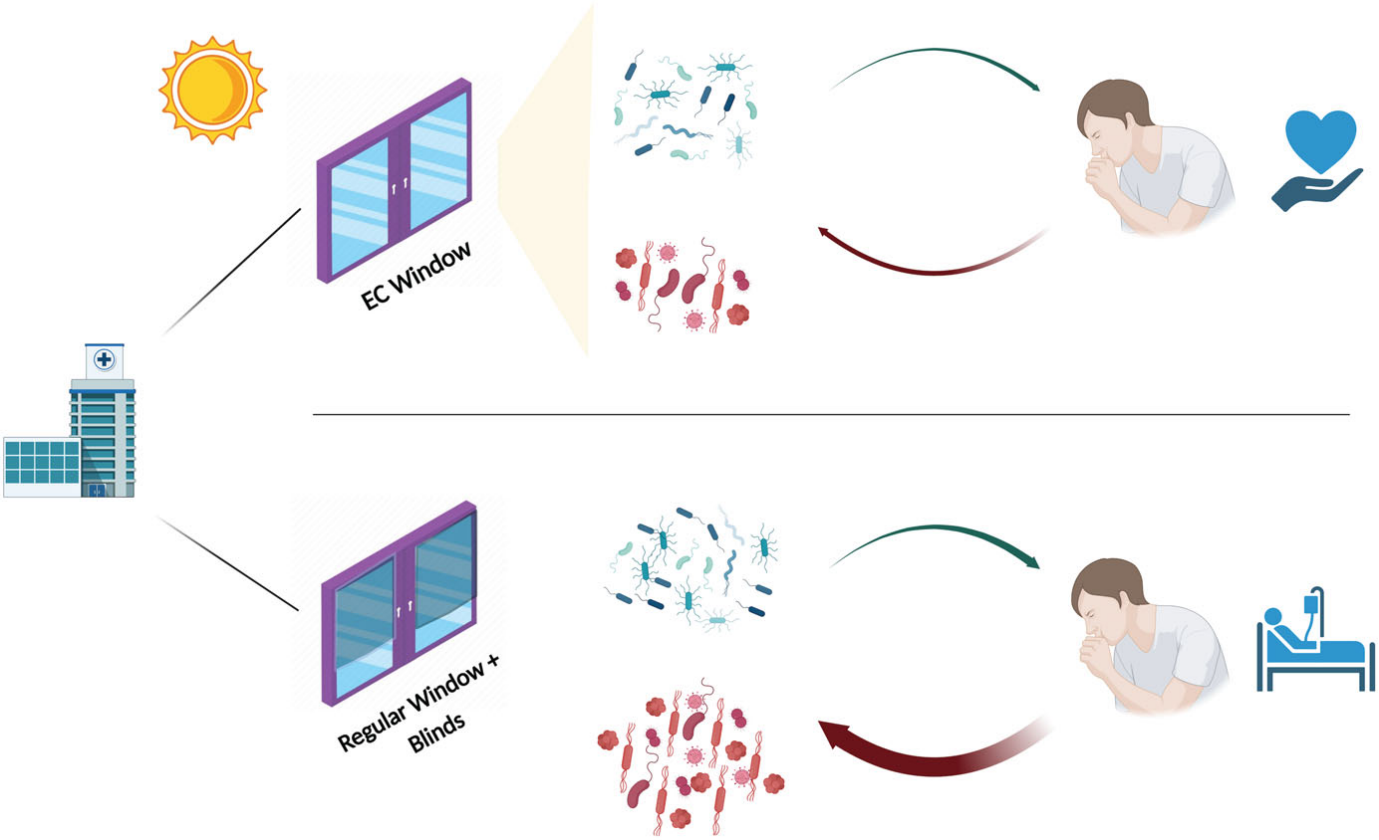
Illustration of indoor daylight through an EC Window (top) and traditional window with blinds (bottom) in a hospital. Occupants and indoor microbiome interact in both directions, which could contain beneficial microbiome (green) and pathogens (red). Bringing more daylight into buildings using an EC window (top) may reduce pathogen growth.

The relative abundance of *Staphylococcus* on window surfaces was lower over the five-day EC window activation compared to baseline (T0). Although many *Staphylococcus* species are commensal bacteria that are non-pathogenic to humans, pathogenic *Staphylococcus* can cause severe infections in the bloodstream, lungs, and soft tissues.^28^ Several strains are also biofilm formers, which can cause nosocomial infections and life-threatening sepsis.^20^ A stochastic compartmental model examined the transmission of *MRSA* in 18 intensive care unit (ICU) also found that the overall acquisition rate of *MRSA* could be reduced by 11%–13% with the use of EC windows, suggesting that such window has the potential to minimize infections especially in healthcare settings (Figure 7). Furthermore, it has been shown that EC windows may promote occupant satisfaction, stress relief, and reduction of anxiety by increasing access to daylight and natural views without the obstruction of blinds.^11–13,29–31^

Several limitations of this study deserve attention: 16s rRNA Illumina sequencing only allows for reliable identification of microorganisms at the genus level, thus we cannot distinguish between pathogenic and non-pathogenic bacteria, especially in the genus ESKAPE. Future studies should consider using other sequencing platforms such as metagenomics to determine the association between EC windows and the abundance of pathogenic bacteria specifically. Additionally, only 10 patient rooms were included in the experiment, with samples collected only up to five days of the patient’s stay. Though no significant changes were observed in the patient’s microbiome, it is possible that longer hospital stays, or larger sample sizes may have resulted in different outcomes. Although patients assigned to Regular Rooms agreed to leave the blinds in the assigned position, it is possible that patients may have altered the blinds without informing the study team. However, we expect that any blind alteration would be non-differential and any resultant bias would be toward the null. We also only recruited Hematology-Oncology patients which is another limitation as the environmental surfaces’ microbiome were largely driven by patient microbiome. The observed microbiome changes hence may not be reflective of other patient populations. Future studies should explore the impact of EC window in other patient units and in other hospitals with different building design to better understand the association of window types and clinical outcomes—particularly the HAI rates.

## Supporting information

Lam et al. supplementary material Lam et al. supplementary material

## Supplementary material

For supplementary material accompanying this paper visit http://doi.org/10.1017/ash.2024.344. click here to view supplementary material

## Research transparency

All codes and raw data for statistical analysis are publicly available at GitHub repository: https://github.com/RitaLam/Microbial-analysis.
