# Supplementary material for: The spatial and temporal effect of electrochromic windows on indoor and human microbiome in an inpatient hospital
Source: Antimicrob Steward Healthc Epidemiol. 2024 Oct 25;4(1):e188. doi: 10.1017/ash.2024.344 (PMC11504557; doi:10.1017/ash.2024.344)
Supplement: Lam et al. supplementary material [file S2732494X24003449sup001.docx]

# **Supplemental Information: Methodology**

***Environmental surface sample collection***

Prior to each surface sample collection, 500μL of sterile 1X Phosphate Buffer Saline (PBS) was prepared in a 1.5ml Eppendorf tube inside the laminar hood. A customized sampling frame (20 x 20cm) was also made for a sampling area of 400cm^2^. At the sampling time, a sterile BD BBL^TM^ CultureSwab was first dipped into the prepared 500μL 1X PBS solution, then swabbed horizontally, vertically, and diagonally within the sampling frame on the surfaces for 30 seconds. The swab tip was then immediately cut with a sanitized scissor and placed into the same Eppendorf tube with the 1X PBS solution. All samples were stored at -80°C freezer immediately upon collection for further analyses.

## ***DNA extraction***

Total DNA from swabs and PTFE air filters was extracted using the DNeasy PowerSoil Kit (Qiagen, USA). Saliva samples were extracted using QIAmp DNA Microbiome Kit (Qiagen, USA). All extractions followed manufacturers’ instructions, with some modifications: to improve yield of extraction, swabbed tips were incubated at 65°C for 10 minutes. FastPrep-24 Powerlyzer (MP Biomedical, USA) was then used for two rounds of homogenization. Air samples collected with the PTFE filters were also incubated inside a sonication bath (40kHz) at 60°C for 30 minutes before extraction.

## ***16s rRNA Illumina sequence preparation***

Due to the low biomass nature of environmental samples, the V3V4 region of 16s rRNA were amplified (341F-806R) with two rounds of polymerase chain reaction (PCR) prior sending for sequencing. The detail of first round of PCR is as follows: reaction mix (20μL) contains 2mM (2μL) of dNTP Mix (Thermo Fisher #R0241), 10μM of 16s V3V4 forwarded primer (341F: 5’-CCTACGGGNGGCWGCAG-3’) (1μL) and reverse primer (805R: 5’-GACTACHVGGGTATCTAATCC -3’) (1μL), 2μL of 10x PCR Buffer, 1μL of 25nM magnesium chloride (MgCl_2_ ), 0.4μL of AmpliTaq Gold DNA Polymerase (Fisher Scientific, Applied Biosystems™ N8080259), 9.6μL of PCR grade water, and 3μL of template DNA. Thermocycling conditions were as follows: Initial denaturation at 95°C for 10min, 35 cycles of 15s at 95°C, 30s at 54°C, 1min at 72°C, and final extension at 72°C for 5min. The amplified PCR products were run on a 2% agarose gel; if PCR was successful and clear bands were obtained, the PCR products were prepared for sequencing. If no bands were observed, second PCR was performed. The 1^st^ PCR product was cleaned with ExoSAP-IT^TM^ (Thermo Fisher Scientific, Canada) to remove excessive primer and nucleotides. Reaction mix for the second PCR was modified as follow: 2mM (2μL) of dNTP Mix, 2μL of 10x PCR Buffer, 10μM of 16s V3V4 (341F-805R) forward primer (0.4μL) and reverse primer (0.4μL), 0.8μL of 25nM of MgCl_2_, 0.4μL of AmpliTaq Gold DNA Polymerase, 13μL of PCR grade water, and 1μL of cleaned PCR product. Thermocycling conditions remained the same as the 1^st^ PCR. The amplified PCR products were run on a 2% agarose gel. Samples with bands (N=176) were sent for 16s Illumina MiSeq sequencing generating 250bp paired end reads.

## ***16s MiSeq illumnia sequencing data processing***

Raw 16s rRNA amplicon sequencing data was processed using Quantitative Insights into Microbial Ecology pipeline (QIIME2) 2022.8 for standard demultiplexing and quality filtering,^1^ Paired end reads had the barcode and primers removed. Forward reads were quality trimmed at 270bp and reverse reads were trimmed at 220bp to ensure a quality score of 20 (< 1% incorrect bases). *DADA2* was the denoising statistical inference algorithm for identification of amplicon sequence variants (ASVs).^2^ Taxonomy was assigned using the SILVA version 132 reference database with sequence identify cut-off set at 99%.^3^ The obtained ASVs were imported and processed using the R version 4.2.0; ASVs identified as mitochondria and chloroplasts were first removed. ASVs with less than 3 reads and less than 0.1% of the total sequences were excluded from further analysis. Relative abundance of ASVs were identified at genus level. A total of 7975 unique ASVs were obtained after QIIME2 analysis. After filtering and sequencing processing, 5092 ASVs were included in the final downstream analysis. The raw sequencing data has been deposited into the National Center for Biotechnology Information (NCBI) Sequence Read Archive (SRA) under BioProject ID PRJNA928218.

## ***Real-time quantitative polymerase chain reaction (qPCR)***

The reaction mix for qPCR was as follows: 5μL of SsoAdvanced Universal SYBR Green Supermix (Bio-Rad, Canada), 0.25μL of each 20μM forward primer 515F (5’-GTGYCAGCMGCCGCGGTA A-3’) and reverse primer 806R (5'-GGA CTA CNV GGG TWT CTA AT-3') (Integrated DNA Technologies, Canada), 3.5μL of PCR grade nuclease free water, and 1μL of DNA template for a total reaction volume of 10μL. The thermocycling conditions were as follows: Initial denaturation for 30s at 95°C, 35 cycles of 15s at 94°C, 30s at 55°C, and 20s at 70°C. Standard curves were generated using a 280bp synthetic gBlocks Gene Fragment~~s~~ (Integrated DNA technology, Canada) based on the 16s rRNA of *Pseudomonas denitrificans*.^4^ A ten-fold dilution series was made from 1 million copies per μL to 10 copies per μL and was plated in triplicate. The abundance of bacterial load for each sample was calculated from the linear regression equation as determined by the standard curve in terms of copy number per μL (**Figure S1**).

## ***Statistical analysis and bioinformatics***

*Environmental data*

To assess the environmental conditions in the Regular Rooms and EC Window Rooms, normality of each dataset was first assessed with Shapiro Wilk test. Since all datasets failed the normality test (Shapiro Wilks test: *P* < 0.05), the Mann-Whitney U test was used to compare each environment parameter (light intensity, temperature, RH, CO_2_, and PM2.5) between Regular Rooms and EC Window Rooms.

*Bacteria source tracking*

To better understand the indoor microbial community, we performed a Bayesian-based source tracking analysis using the SourceTracker 2 classifier^5^ to determine the contribution of patient microbiome as sources of indoor microbiome communities.

After obtaining all ASVs at the genus level, a composite ASV table of source samples (palm and saliva) and sink samples (window, blinds, and air samples) was used as the input for the SourceTracker2 algorithm. The relative contribution of each source sample was averaged for each sink sample and presented in a pie chart.

*Microbial diversity and community composition*

For microbial diversity and community analyses, indices were calculated and analyzed in R version 4.2.0. Following McCurdie and Holmes,^6^ ASVs were not rarefied and transformed into proportion (counts divided by total library sides). To assess the alpha diversity, Shannon index was calculated using the vegan R package. Following the normality and variance assessment, Shannon indices were compared between the Regular Room and EC Window Room groups using either a t-test (Shapiro Wilks test: *P* > 0.05, Levene’s test: *P* < 0.05) or Mann Whitney-U test (Shapiro Wilks test: *P* < 0.05 or Levene’s test: *P* > 0.05). Beta diversity was tested with weighted UniFrac, unweighted UniFrac and Bray-Curtis dissimilarities. All distance matrices were generated in R using the phyloseq package. Weighted UniFrac was chosen as the distance matrix for Principal coordinate analysis (PCoA) and permutational multivariate analysis of variance (PERMANOVA) to assess the similarity and dissimilarity of bacteria communities.

*Absolute bacterial abundance (qPCR)*

For qPCR total abundance data, the normality and variance of each dataset was first assessed using Shapiro-Wilks test and Levene’s test. If the data followed normal distribution (Shapiro Wilks test: *P* > 0.05) and the assumption of homogeneity of variance were met (Levene’s test: *P* < 0.05), t-tests were performed to compare results between the Regular Room and EC Window Room groups. If either normality or variance test failed, Mann-Whitney-U tests were performed. All statistical tests were performed in Python version 3.8.13 with SciPy package 1.9.3.

*Relative abundance of genus ESKAPE pathogens*

After obtaining the relative abundance of bacteria at the genus level, statistical test was performed on genus identified under *Enterococcus*, *Staphylococcus,* *Klebsiella*, *Actinobacteria*, *Pseudomonas* and *Enterobacter.* T-test (both normality and variance test were passed) or Mann-Whitney U test (either normality or variance assumption failed) were performed to compare the results between Regular Room and EC Window Room groups. Pairwise comparison with Tukey’s honest significant difference (HSD) test was also performed to compare the results between time in Regular Room and EC Window Room groups to see the effect of EC window over the first five-days of patient stay.

**REFERENCES**

1. Bolyen E, Rideout JR, Dillon MR, et al. Reproducible, interactive, scalable and extensible microbiome data science using QIIME 2. *Nature Biotechnology 2019 37:8*. 2019;37(8):852-857. doi:10.1038/s41587-019-0209-9

2. Callahan BJ, McMurdie PJ, Rosen MJ, Han AW, Johnson AJA, Holmes SP. DADA2: High resolution sample inference from Illumina amplicon data. *Nat Methods*. 2016;13(7):581. doi:10.1038/NMETH.3869

3. Quast C, Pruesse E, Yilmaz P, et al. The SILVA ribosomal RNA gene database project: improved data processing and web-based tools. *Nucleic Acids Res*. 2013;41(D1):D590-D596. doi:10.1093/NAR/GKS1219

4. Masrahi A, Somenahally A, Gentry T. Interactions of Arbuscular Mycorrhizal Fungi with Hyphosphere Microbial Communities in a Saline Soil: Impacts on Phosphorus Availability and Alkaline Phosphatase Gene Abundance. *Soil Systems 2020, Vol 4, Page 63*. 2020;4(4):63. doi:10.3390/SOILSYSTEMS4040063

5. Knights D, Kuczynski J, Charlson ES, et al. Bayesian community-wide culture-independent microbial source tracking. *Nat Methods*. 2011;8(9):761. doi:10.1038/NMETH.1650

6. McMurdie PJ, Holmes S. Waste Not, Want Not: Why Rarefying Microbiome Data Is Inadmissible. *PLoS Comput Biol*. 2014;10(4):e1003531. doi:10.1371/JOURNAL.PCBI.1003531

# **Supplemental Information: Results**

**Table S1** PERMANOVA of beta diversity of window samples based on weighted UniFrac dissimilarities. Analyses compared bacterial communities between Regular Room and EC Window Room with 999 permutations. Significant was indicated (*, *P* < 0.05; **, *P* < 0.01; ***, *P* < 0.001).

| **Time** | **Variable** | **Weighted UniFrac** | | | |
| --- | --- | --- | --- | --- | --- |
|  |  | **Sum Sqrs** | ***R^2^*** | ***F*** | **Pr(>F)** |
| T0 | Room Type | 0.045 | 0.081 | 0.529 | 0.836 |
| T1 | Room Type | 0.339 | 0.255 | 2.736 | 0.007 ** |
| T3 | Room Type | 0.086 | 0.136 | 1.102 | 0.332 |

| 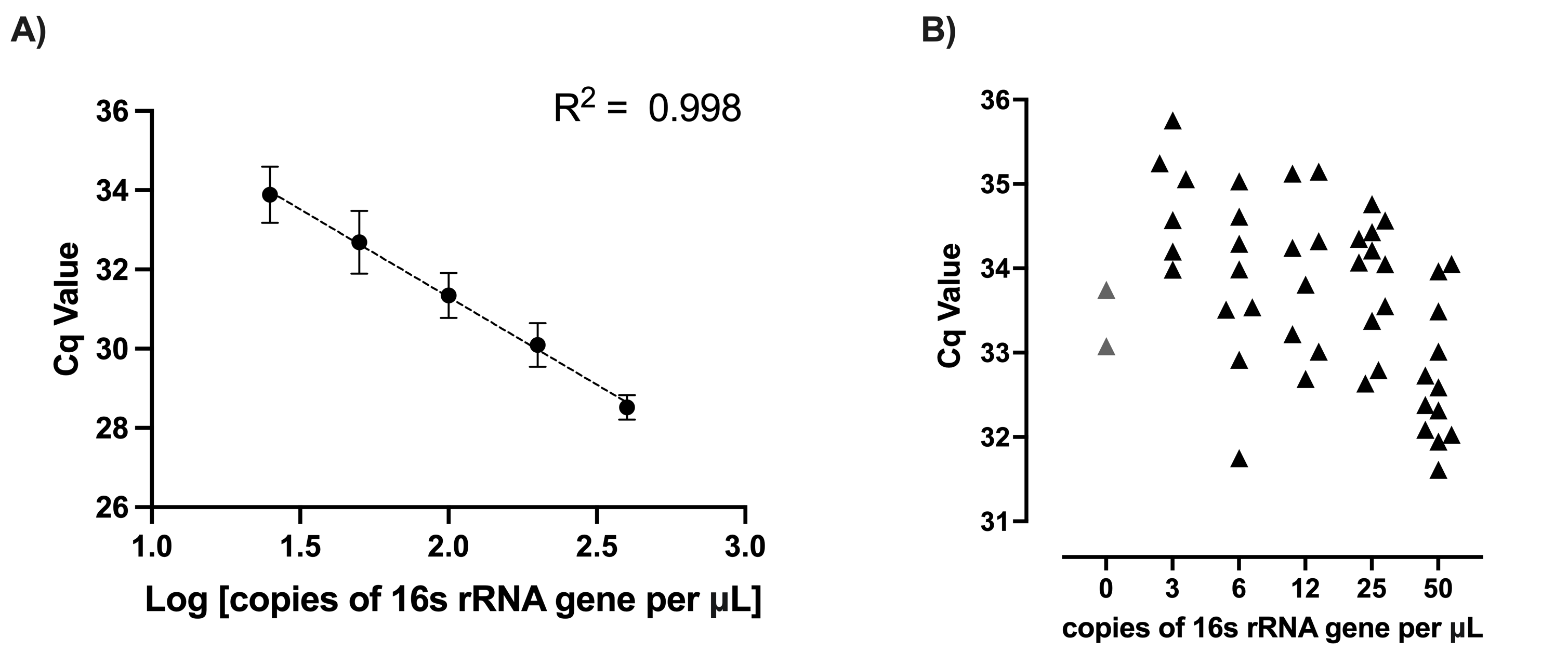 | |
| --- | --- |
| **Standard curve** | y= -4.425x + 40.158 |
| **Negative control (NC) Cq Value (mean ± SD)** | 33.411 ± 0.473 |
| **Limit of detection based on NC** | 33.651 copies of genes per µL |

**Figure S1** Sensitivity of the qPCR assay for absolute bacterial abundance quantification. **A)** Standard curve generated with gBlock with known copies of 16s rRNA genes. Data shows the mean ± SD. **B)** RT-qPCR machine limit of detection (LOD) for the 16s rRNA V4 primer-probe set. The average Cq value of the negative controls (water) is shown at the table below, which based on the generated standard curve is approximately 34 copies of genes.

**
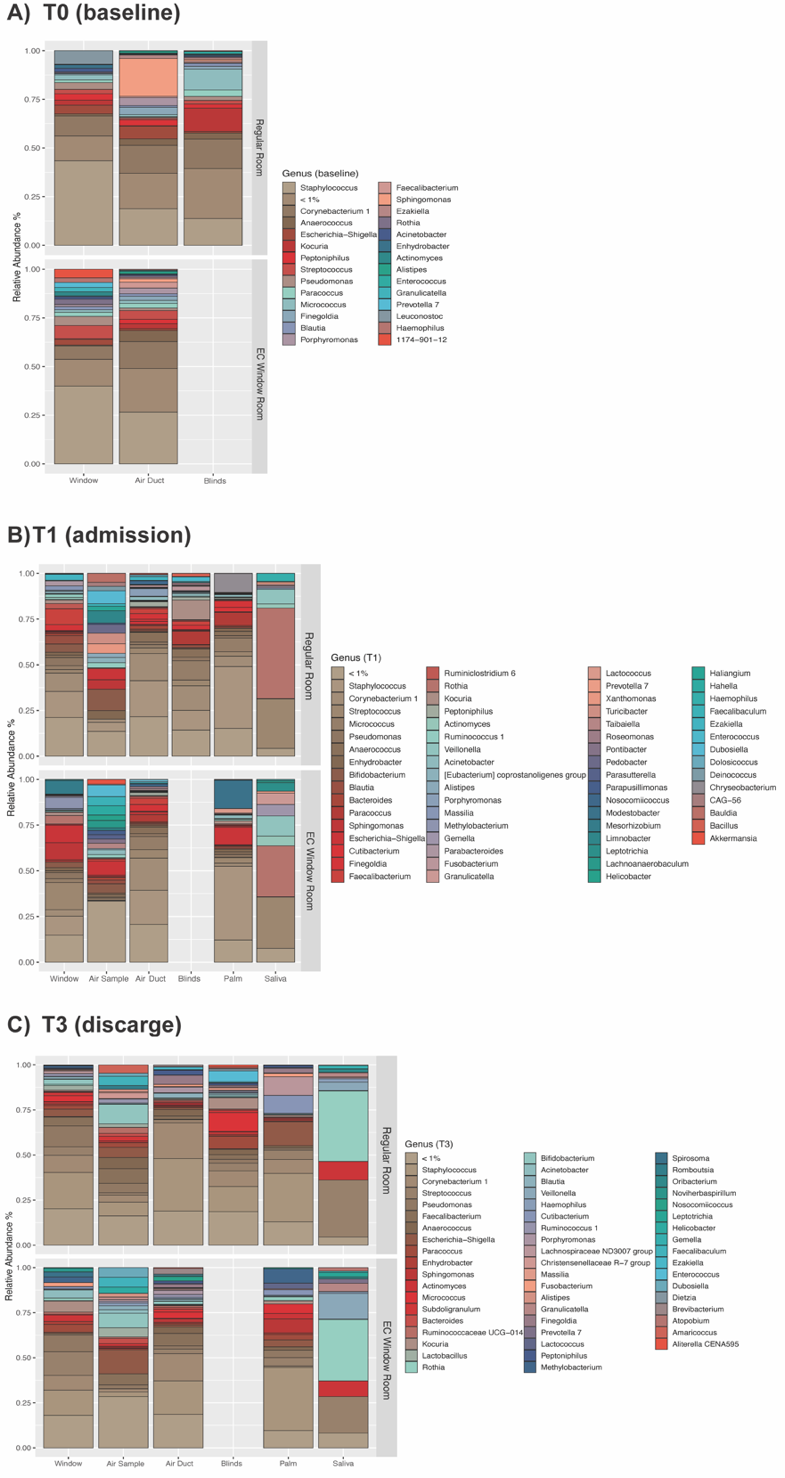
**

**Figure S2** Taxa composition of environmental samples at **A)** T0 (baseline), **B)** T1 (admission), and **C)** T3 (discharge). Regular Room group is shown on the top and EC Window Room group is shown at the bottom. Genus with >1% abundance is shown. Legend is sorted based on abundance, with the most abundant bacteria genera shown on the top left, and the least abundant genera shown on the bottom right.

**
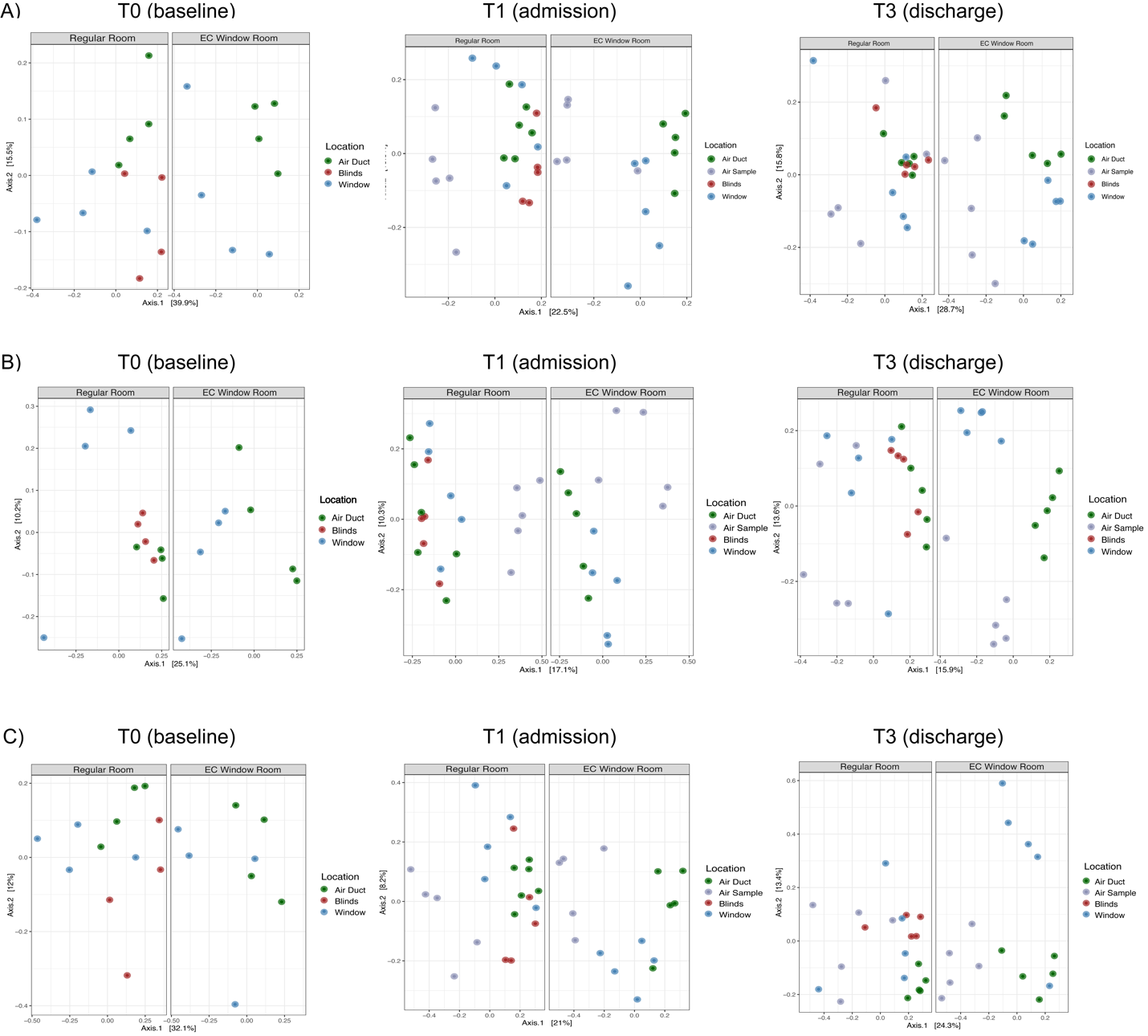
**

**Figure S3** Principal coordinate analysis (PCoA) of beta diversity of environmental samples measured by three dissimilarity distance matrices. **A)** Weighted UniFrac distance matrix. **B)** Unweighted UniFrac distance matrix. **C)** Bray-Curtis distance matrix. Air duct is shown in green; air sample is shown in light purple, blind is shown in red, and window is shown in blue. Regular Room group is shown on the left and EC Window Room group is shown on the left for each PCoA.

**
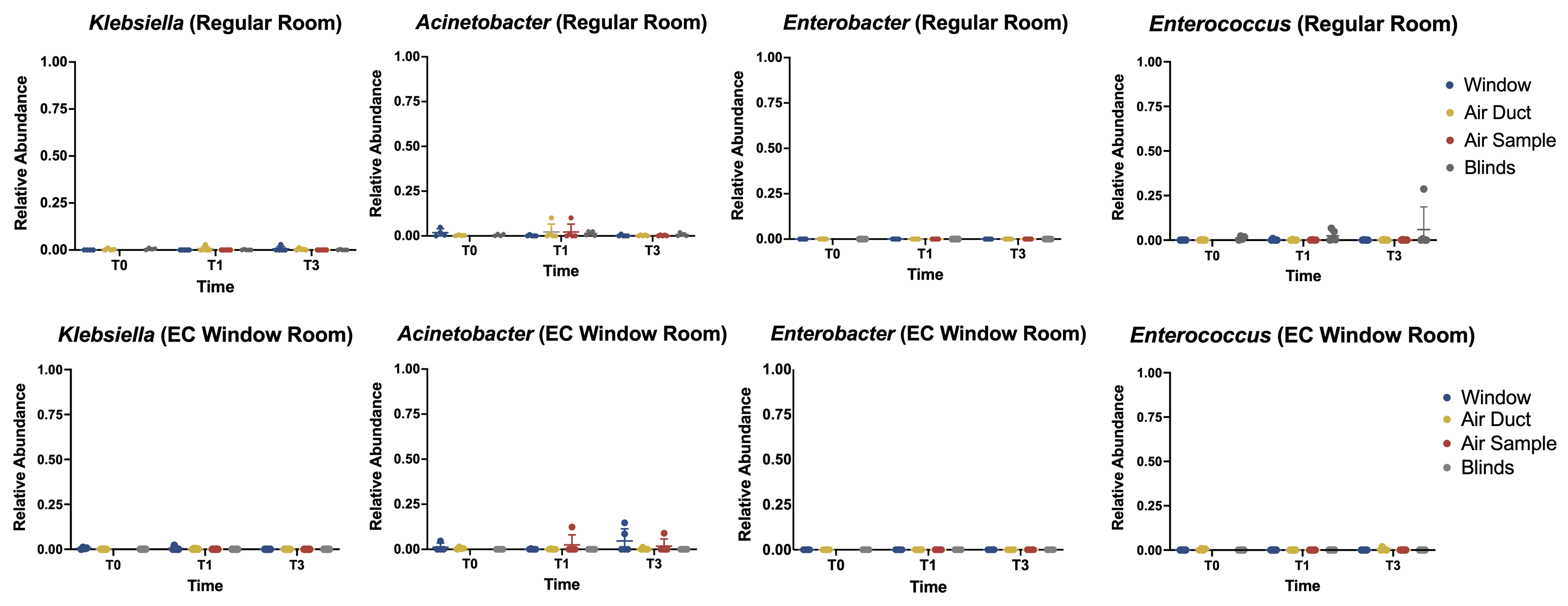
**

**Figure S4** Relative abundance of *Klebsiella, Acinetobacter, Enterobacter,* and *Enterococcus* in environmental samples. Regular Room group is shown at the top and EC Window Room group is shown at the bottom. Window is shown in blue; air duct is shown in yellow; air sample is shown in red, and blind is shown in grey. Data shows the mean ± SEM.
